# Severe Vertex Epidural Hematoma in a Child: A Case Report of a Management without Expert Neurosurgical Care

**DOI:** 10.1155/2011/476416

**Published:** 2011-09-29

**Authors:** Christophe Brévart, Antoine Bertani, Hassan Abdourahman Aden, Paul Menguy, Renaud Dulou

**Affiliations:** ^1^Department of Surgery, HMC Bouffard, SP 85024, 00812 Armées, Djibouti, Djibouti; ^2^Department of Neurosurgery, HIA Val de Grâce, Paris, France

## Abstract

Vertex epidural hematomas (VEDHs) are an uncommon situation and difficulties may be encountered in their diagnosis and management. This is more complicated when the surgical management has to be performed by general surgeons, not specialized in neurosurgery, in a remote location. It was in this context that we were brought to care in charge a 2-year-old boy who required a neurosurgical emergency rescue for a severe VEDH in Djibouti. Through the description of this case, we want to emphasize the value of developing a network of teleconsultation for the benefit of remote and isolated locations and learning basic techniques of emergency neurosurgery.

## 1. Introduction

Epidural hematomas are defined as accumulation of blood between skull and dura mater. They may represent a possibly deadly situation requiring neurosurgical advice. They are invariably associated with head trauma and in the pediatric population have been shown to be present in almost 30% of severe traumatic brain injuries. The common mechanism of injury is falling from a height [1]. Vertex epidural hematomas account for a small portion of all intracranial hematomas. They usually result from vertex fractures which can injure the superior sagittal sinus. Symptoms can be acute, subacute, or chronic in nature and may be related to venous hypertension. Surgical management of such injuries can be quite dangerous if the superior sagittal sinus is involved. Some authors have reported spontaneous resolution without surgical intervention. Others have required burr hole drainage alone. Given the potential for significant blood loss, most authors recommend surgical intervention only for those cases with severe neurologic deterioration or ominous clinical findings [2]. 

This case describes a two-years-old child who presented with a huge bilateral vertex epidural hematoma related to a superior longitudinal sinus lesion, managed by general surgeons, not specialized in neurosurgery, in a remote major hospital in Djibouti [3, 4].

## 2. Case Report

A 2-year-old boy felt down from a balcony (4 m) with direct head trauma and initial brief loss of consciousness. He presented with immediate vomiting and the appearance of a secondary stupor. His family transferred him to the hospital less than an hour after the trauma. 

On arrival, clinical examination revealed an initial Glasgow Coma Scale (GCS) score of 13 with a large right frontoparietal cephalhematoma overlying the right upper eyelid. He presented with tetraparesis, absent tendon reflex, plantar Babinski cutaneous reflex indifferent bilaterally, the left pupil was intermediate and reactive, and eyelid occlusion by the hematoma made evaluation of the right pupil impossible.

**Figure 1:**
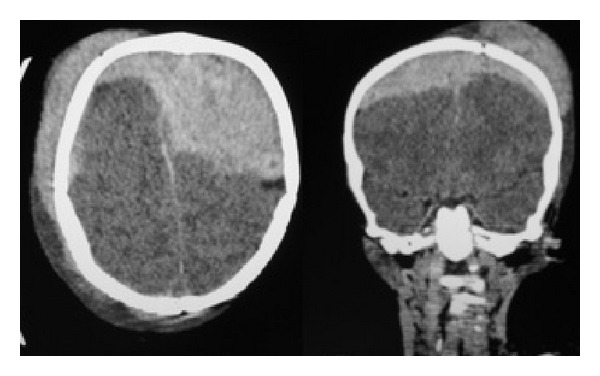
Axial and coronal CT scan images showing vertex epidural haematoma in preoperative course.

**Figure 2:**
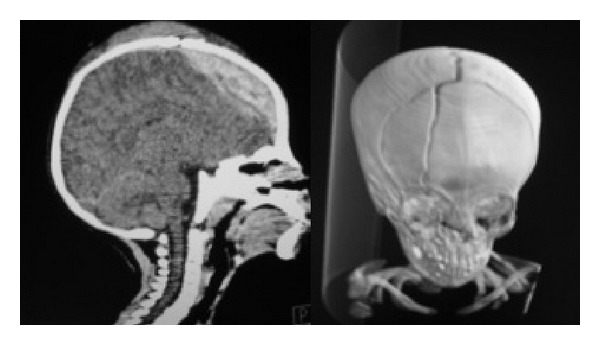
Sagittal and 3D CT scan images showing VEDH and frontal paramedian fracture extending down to the right orbital ceiling.

**Figure 3:**
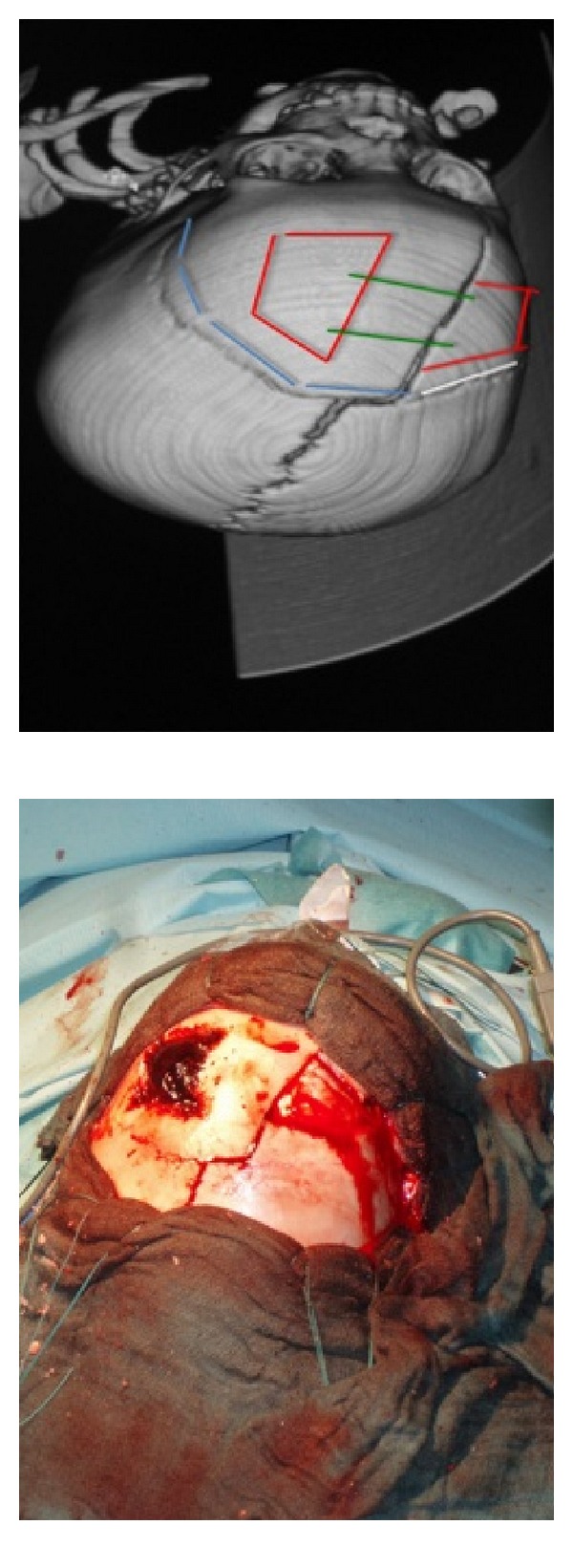
Scheme of incision for operative course and peroperative view.

A selection of images from a noncontrast head CT scan was sent via Personal E-mail to a nonpediatric neurosurgeon after phone contact. Diagnosis was suspected with the characteristic biconvex chap and significant compression and with arguments for VEDH that they usually result from trauma in younger children even if in this case epidural hematoma does cross-cranial sutures. The consultant confirmed the diagnosis of epidural hematoma of the arch (Figures 1 and 2), associated with a fractured frontal paramedian extending down to the right orbital ceiling [5]. There was no associated cervical spine injury. The need for emergent surgical decompression [6] was determined less than 15 minutes after the initial phone call to the neurosurgeon. Surgical technique was discussed via phone transmission and optimized with a scheme (Figure 3) using Microsoft PowerPoint showing skin incision, bilateral craniotomies, and dural tack up sutures crossing the midline. 

**Figure 4:**
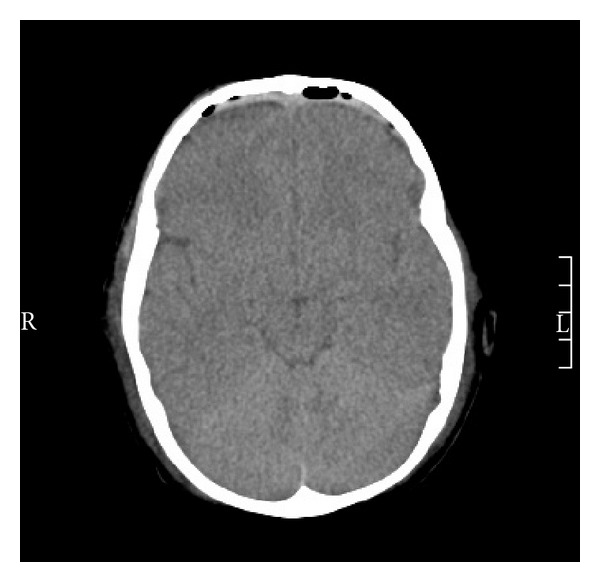
Postoperative control axial CT scan.

We proceeded to the surgery and made a coronal incision following coronal suture, keeping the proposed approach. Raising the skin flap anteriorly exposed the fracture site. Two free bone flaps were performed, from each side of the median line, with Gigli saw and using the fracture line on the right side. The epidural clot was carefully removed. On the left side, this approach was suddenly accompanied with active bleeding probably from venous origin, controlled by tack-up sutures as planned by the scheme preoperatively. The flaps were repositioned with transosseous sutures after dural suspension device. During the postoperative course, the patient showed dramatic improvement and the child was discharged home shortly after repeat control CT Scan (Figure 4). The child recovered *ad integrum *on neurological examination. The only complication was pulp necrosis of the fifth right toe secondary to hemodynamic instability requiring introduction of pressor drugs in postoperative course. 

## 3. Discussion

Most hospitals rely on axial CT scanning as the primary initial imaging modality for patients with potentially intracranial lesions after head injury. Because the vertex epidural hematoma lies in the same plane as the image on axial scanning, it could easily be missed or underestimated [5]. Coronal and sagittal CT scan images provide better information about localization and size of the hematoma. When there is evidence for vertex skull fractures on axial CT, vertex epidural hematoma should be suspected. Magnetic resonance imaging (MRI) may be helpful, with coronal and sagittal sequences but is not readily available in Djibouti [5]. Moreover, it is not routinely performed in emergency in practice. In our case, the volume of the vertex epidural hematoma was atypical in the pediatric setting and required emergency surgery.

Djibouti health conditions are critical: there is no neurosurgeon, no radiologist but two computerised tomography machines, one of them in the French Military Bouffard Hospital, where there is a general surgical team (one general, one orthopaedic surgeon and two anaesthetists). Images are theoretically sent for radiological advice to France. In practice, difficulties encountered with internet facilities limiting us to send CT exams only when necessary. Medical evacuation is possible for the richest patients, but it is impossible in an emergent situation as we have described.

The telecommunication means are composed of classic solutions like internet and telephone, it does not include a special canal for telemedicine. A discussion was started with the neurosurgeon, known personally by the two surgeons, of the French Army Hospital of Val-de-Grâce for the surgical management after the transfer of a selection of CT scan by Internet. The lack of telemedicine channel is a high difficulty, in a remote major hospital in Africa, because the transfer is very long and we cannot permit to go slowly by a 2-year-old child. That is why, it is necessary to realize a good selection of CT scan images sending by Internet in association with a phone conference. The good results of surgery is probably the combination of fast answer by neurosurgeon, with cleverness for incision (Figure 3) and peroperative management, and the learning and training's management of neurotrauma by general surgeons in the French Army. If there is no Internet connection available, use of military satellite link technology is impossible, because any bandpass is intended for medical applications. Thus, phone connection remains the only solution.

There is a considerable discrepancy between the potential demand for neurosurgeons and the actual availability of such specialists not only in civilian settings but even more so in a poor country of East Africa like Djibouti. In civilian settings, generalist surgeons have become obsolete and are being replaced by specialists for all branches of surgery [3]. The Medical French Army conducts courses for surgeons and orthopedists on the management of neurotrauma. That is why general surgeons are no longer required to learn how to perform a craniotomy. In the Medical French Army, we have conserved the possibility to acquire this performance thanks to practice above all in pig and human cadavers. These two surgeons have successfully used their neurosurgical knowledge in countries under development and provided surgical treatment to patients with traumatic brain injuries.

## 4. Conclusion

This case showed the efficiency of the combination of phone and Internet links coupled with the experience of nonneurosurgeons to manage an emergency neurosurgical situation in a remote area [7]. Advances in Internet facilities and specific formations for general surgeons to neurosurgical procedures deployed in Djibouti seem to be essential to improve these managements, since no neurosurgical department is planned in the civilian principal sanitary structure for the near future in this country.

In these situations, it was particularly helpful when the physicians deployed abroad and the neurosurgeon in France knew each other personally. In the future, efforts will be made to combine telemedicine [7] and neuronavigation in an attempt to further improve direct support for physicians under military deployment conditions.
